# Urinary Diversion–Specific Morbidity After Radical Cystectomy: A Ten‐Year Institutional Experience

**DOI:** 10.1002/cam4.71684

**Published:** 2026-03-15

**Authors:** Xuanhan Hu, Jia Miao, Yueyu Huang, Yunkai Yang, Xinyu Zhang, Yifan Wang, Lin Qian, Dahong Zhang, Haibin Wei

**Affiliations:** ^1^ Urology & Nephrology Center, Department of Urology Zhejiang Provincial People's Hospital (Affiliated People's Hospital, Hangzhou Medical College) Hangzhou China; ^2^ The Second School of Clinical Medicine Zhejiang Chinese Medical University Hangzhou China

**Keywords:** bricker ileal conduit, cutaneous ureterostomy, orthotopic neobladder, postoperative complications, radical cystectomy, urinary diversion

## Abstract

**Introduction:**

This study investigates the correlations and risk factors of complications associated with different urinary diversion methods after radical cystectomy (RC) for bladder cancer (BC).

**Methods:**

A retrospective analysis was conducted on 574 bc patients treated between 2012 and 2022. Complications were categorized as early or late occurrences. Multistate Cox regression and stepwise logistic regression models were employed to identify independent predictors. Heat maps were utilized to explore correlations among complications.

**Results:**

Patients undergoing ureterostomy were generally older, had a higher prevalence of comorbidities, and exhibited a greater propensity for late urinary tract infections (UTIs), nephrolithiasis, and anxiety/depression. Bricker conduits were linked to small‐bowel obstruction and ureteroenteric strictures, while orthotopic neobladders were associated with incontinence and urinary retention. Diabetes increased risks of urolithiasis and mild bowel obstruction but decreased strictures and reflux. High pathological grade predicted strictures; low hemoglobin increased obstruction and late UTIs. Robot‐assisted laparoscopy reduced early UTIs, reflux, and ostomy‐related obstruction. Bowel obstruction risk was elevated in patients with higher body mass index or smoking history but was mitigated by robotic approaches. Late UTIs were strongly linked to ureterostomy and heavy smoking.

**Conclusion:**

Ureterostomy raises the risk of UTIs, kidney issues, and psychological disorders, necessitating careful follow‐up. Bricker conduits require monitoring for bowel complications, while orthotopic neobladders are linked to incontinence and metabolic problems, demanding careful patient selection. Advanced age, heavy smoking, T4 stage, and long hospital stays are key predictors of complications and should guide preoperative risk assessment. Robot‐assisted laparoscopy lessens gastrointestinal and stoma‐related events.

## 1 Introduction

Radical cystectomy (RC) is the standard surgical treatment for muscle‐invasive bladder cancer (MIBC) [1]. With advances in surgical techniques, urinary diversion methods have increasingly diversified, aiming to improve postoperative quality of life. The main types of urinary diversion currently employed include ureterocutaneostomy, Bricker ileal conduit, and orthotopic neobladder [2]. However, each method carries specific complication risks, which can significantly affect patient prognosis and quality of life [3, 4].

Ureterocutaneostomy is a relatively simple method of urinary diversion, but it is often associated with long‐term complications such as stoma stenosis and infections [5]. The Bricker ileal conduit is another commonly used option; however, it carries risks of postoperative complications such as bowel obstruction and anastomotic leakage [6]. Additionally, patients must wear an external ostomy bag permanently, which may negatively impact body image and social interaction [7]. To enhance quality of life, orthotopic neobladder reconstructions, such as U‐shaped and W‐shaped orthotopic neobladders, have been developed [8]. These approaches aim to restore near‐normal voiding function, but they are also associated with increased risks of urinary incontinence, urinary retention, and metabolic disturbances.

In recent years, a growing number of studies have investigated the impact of different urinary diversion techniques on postoperative complications [9, 10]. Pyrgidis et al. compared ileal conduits and orthotopic neobladders in terms of renal function, metabolic outcomes, and health‐related quality of life, finding that neobladder recipients experienced fewer metabolic disturbances and higher global quality of life (QOL) scores [11]. Hassan et al.'s prospective evaluation of a modified Y‐shaped neobladder demonstrated a significant reduction in ureteroileal anastomotic strictures (3% vs. 12%, *p* = 0.04) and improved daytime continence rates (94% vs. 88%, *p* = 0.03), all without detriment to 5‐year overall or cancer‐specific survival [12]. Moreover, patient‐specific factors such as age, comorbidities, and prior abdominal surgeries significantly influence both the choice of diversion and the risk of adverse events, with elderly or high‐risk patients more likely to undergo simpler conduits to minimize operative stress [13].

Building upon the existing body of literature, including recent studies on perioperative or in‐hospital outcomes after radical cystectomy, the present study retrospectively analyzes a decade of clinical data from our institution to investigate the association between urinary diversion techniques and postoperative complications following RC for bladder cancer (BC). By evaluating the incidence and characteristics of complications associated with each urinary reconstruction method, this study aims to identify technique‐specific complication profiles and provide clinically meaningful insights into the risk–benefit balance of different diversion strategies. Ultimately, our findings may inform surgical decision‐making and contribute to the optimization of individualized treatment plans, thereby enhancing long‐term outcomes and quality of life for patients undergoing RC.

## 2 Materials and Methods

### 2.1 Study Design

The study was conducted at the Department of Urology, Zhejiang Provincial People's Hospital, People's Hospital of Hangzhou Medical College, Hangzhou, Zhejiang, China. All patients presenting with BC and undergoing RC at our institution between January 1, 2012, and December 31, 2022, were retrospectively reviewed. All participants were fully informed about the planned procedures and provided informed consent prior to inclusion. The study protocol was approved by the Ethics Committee of Zhejiang Provincial People's Hospital. All procedures were carried out in accordance with the Declaration of Helsinki and its subsequent amendments.

The inclusion criteria were as follows: (1) histologically confirmed diagnosis of MIBC or non‐muscle‐invasive bladder cancer (NMIBC) with multiple recurrences and a strong patient preference for RC; (2) patients who underwent RC combined with urinary diversion; (3) availability of complete perioperative and follow‐up clinical data.

The exclusion criteria were as follows: (1) patients with incomplete clinical data or lost to follow‐up; (2) those with confirmed distant metastases prior to surgery; (3) patients with preoperative severe renal insufficiency (eGFR < 30 mL/min/1.73 m^2^) or requiring dialysis; (4) individuals with a history of pelvic radiotherapy or major pelvic surgery that could significantly affect surgical outcomes; (5) those with coexisting malignancies requiring systemic treatment. The detailed patient selection process, including exclusions due to missing key variables and loss to follow‐up, is illustrated in the study flow diagram (Figure S1).

### 2.2 Surgeon Experience and Learning Curve

In our department, three specialized surgical teams are dedicated to the treatment of BC. All surgeons involved in this study had completed the required institutional training and had surpassed the established learning curve (defined as a minimum of 30 independently performed cases) for laparoscopic radical cystectomy (LRC), robot‐assisted radical cystectomy (RARC), and various urinary diversion techniques. This ensured surgical proficiency across all procedures and minimized potential bias related to the learning curve effect.

### 2.3 Therapeutic Regimen

Patients underwent thorough preoperative evaluations and were assessed for surgical risk using the American Society of Anesthesiologists (ASA) classification. Once surgery‐ready, the type of urinary diversion (ureterocutaneostomy, Bricker ileal conduit, or orthotopic neobladder) was chosen collaboratively by the patient and surgeon. During surgery, diversions followed standardized protocols with ureteric stents placed at anastomoses. Postoperatively, catheters were used for bladder irrigation or conduit patency and removed on days 3–5 for conduits or day 10 for neobladders after checking anastomotic integrity. Follow‐ups at 3, 6, and 12 months, then annually, assessed renal function, imaging, diversion outcomes, and QOL, with all complications recorded. Postoperative complications were prospectively recorded and classified as early (≤ 30 days) or late (> 30 days) after RC [14].

### 2.4 Statistical Analysis

Baseline, intraoperative, and pathological variables were compared across urinary diversion groups using one‐way analysis of variance or Kruskal–Wallis tests for continuous variables and χ^2^ or Fisher's exact tests for categorical variables, with Bonferroni‐corrected post hoc comparisons applied as appropriate. Correlations among postoperative complications were explored using correlation heatmaps.

Time‐to‐event analyses were performed using a multistate Cox proportional hazards model to evaluate transitions from the initial postoperative state to early complications (≤ 30 days) and subsequently to late complications (> 30 days), with the date of radical cystectomy defined as time zero. Patients were censored at the time of last follow‐up or death if no transition occurred. Cumulative incidence curves were constructed to visualize the timing of complication states. Stepwise multivariable logistic regression analyses were further conducted to identify independent predictors of specific complications. Patients with missing key covariates or incomplete follow‐up were excluded from the corresponding analyses, and complete‐case analyses were applied. All statistical analyses were performed using R software version 4.1.1, with two‐sided *p* values < 0.05 considered statistically significant.

## 3 Results

### 3.1 Demographic Characteristics of Patients

A total of 574 patients who underwent urinary tract reconstruction at Zhejiang Provincial People's Hospital were included in the study. Among them, 192 patients received Bricker ileal conduit, 138 patients underwent U‐shaped orthotopic neobladder reconstruction, 30 patients received W‐shaped orthotopic neobladder, and 214 patients underwent cutaneous ureterostomy.

Table 1 summarizes and compares the baseline demographic and clinical characteristics across the four groups. Statistically significant differences were observed in age, gender, body mass index (BMI), occupation, daily cigarette, chronic kidney disease history of abdominal surgery, and retirement status among the groups. Patients undergoing cutaneous ureterostomy were significantly older than those in the other groups (*p* < 0.001). Body mass index was higher in patients with U‐ or W‐shaped orthotopic neobladders compared to those receiving Bricker or cutaneous ureterostomy (*p* = 0.020). The retirement rate was significantly lower in the U‐shaped neobladder group than in the Bricker ileal conduit and cutaneous ureterostomy groups. Cigarette consumption was highest in the U‐shaped neobladder group, showing significant differences across the cohorts (*p* = 0.010). Additionally, heart disease was more prevalent in the cutaneous ureterostomy group (*p* = 0.037). These differences were further clarified through pairwise comparisons indicated by superscript letters in the table footnotes.

**TABLE 1:** Baseline characteristics of patients stratified by type of urinary tract reconstruction.

Characters	Bricker ileal conduit	U‐shaped orthotopic neobladder	W‐shaped orthotopic neobladder	Cutaneous ureterostomy	*p*
*n*	192	138	30	214	
Age (years, median [IQR])^a^, ^c^, ^e^, ^f^	77.00 [70.00, 83.00]	73.50 [65.00, 81.00]	74.50 [67.75, 80.75]	82.50 [75.25, 88.00]	< 0.001
Body mass index (kg/m^2^, median [IQR])^f^	22.30 [20.60, 24.92]	23.45 [20.93, 25.92]	23.55 [21.92, 25.92]	22.49 [20.22, 24.23]	0.020
Gender (%)^e^					0.064
Female	44 (22.9)	18 (13.0)	4 (13.3)	49 (22.9)	
Male	148 (77.1)	120 (87.0)	26 (86.7)	165 (77.1)	
Occupation (%)^b^					0.519
Cadre	18 (9.4)	11 (8.0)	3 (10.0)	18 (8.4)	
Doctor	1 (0.5)	3 (2.2)	2 (6.7)	5 (2.3)	
Farmer	35 (18.2)	26 (18.8)	4 (13.3)	30 (14.0)	
Freelancer	5 (2.6)	6 (4.3)	0 (0.0)	8 (3.7)	
Staff	62 (32.3)	38 (27.5)	7 (23.3)	63 (29.4)	
Teacher	3 (1.6)	6 (4.3)	3 (10.0)	8 (3.7)	
Worker	68 (35.4)	48 (34.8)	11 (36.7)	82 (38.3)	
Retirement (%)^a^, ^e^, ^f^	186 (96.9)	120 (87.0)	27 (90.0)	211 (98.6)	< 0.001
Smoking history (%)	73 (38.0)	52 (37.7)	9 (30.0)	84 (39.3)	0.825
Smoking cessation (%)	26 (13.5)	25 (18.1)	3 (10.0)	35 (16.4)	0.586
Drinking history (%)	45 (23.4)	28 (20.3)	7 (23.3)	35 (16.4)	0.316
Alcohol abstinence (%)	11 (5.8)	11 (8.0)	2 (6.7)	14 (6.5)	0.878
Daily cigarette (*n*, median [IQR]^c^, ^d^, ^e^)	10 [5, 20]	18 [10, 20]	10 [5, 20]	10 [5, 20]	0.010
Daily alcohol consumption (mL, median [IQR])	50 [30, 150]	100 [40, 150]	50 [35, 125]	50 [45, 100]	0.799
Diabetes (%)	25 (13.0)	13 (9.4)	3 (10.0)	31 (14.5)	0.569
Hypertension (%)	43 (22.4)	29 (21.0)	3 (10.0)	56 (26.2)	0.222
Heart disease (%)	8 (4.2)	3 (2.2)	1 (3.3)	19 (8.9)	0.037
Chronic kidney disease (%)^e^	6 (3.1)	2 (1.4)	0 (0.0)	5 (2.3)	0.796
History of abdominal surgery (%)^e^	36 (18.8)	15 (10.9)	5 (16.7)	45 (21.0)	0.087
Median follow‐up time (months, median [IQR])	34 [22–56]	41 [28–65]	32 [20–54]	38 [25–60]	0.218

Abbreviation: IQR, interquartile range.

^a^ Indicates a statistically significant difference between Bricker ileal conduit and U‐shaped orthotopic neobladder (*p* < 0.05).

^b^ Indicates a statistically significant difference between Bricker ileal conduit and W‐shaped orthotopic neobladder (*p* < 0.05).

^c^ Indicates a statistically significant difference between Bricker ileal conduit and cutaneous ureterostomy (*p* < 0.05).

^d^ Indicates a statistically significant difference between U‐shaped orthotopic neobladder and W‐shaped orthotopic neobladder (*p* < 0.05).

^e^ Indicates a statistically significant difference between U‐shaped orthotopic neobladder and cutaneous ureterostomy (*p* < 0.05).

^f^ Indicates a statistically significant difference between W‐shaped orthotopic neobladder and cutaneous ureterostomy (*p* < 0.05).

### 3.2 Surgical Characteristics and Follow‐Up Outcomes

Table S1 shows the surgical and pathological characteristics of patients who underwent different types of urinary tract reconstruction. Most surgeries were performed using laparoscopy in all groups, with no major difference in surgical method. High‐grade urothelial carcinoma was the most common tumor type. There were no significant differences in prostate cancer occurrence, tumor stage, nerve or vascular invasion, although the W‐shaped group showed a slightly higher rate of vascular invasion. The drop in hemoglobin after surgery was similar across the groups. However, hospital stays were longer in patients with U‐shaped and W‐shaped orthotopic neobladders compared to those with Bricker ileal conduits or cutaneous ureterostomies (*p* = 0.020). The median postoperative length of stay was 11 days (IQR 9–13.5) for Bricker ileal conduit, 12 days (IQR 10–14) for U‐shaped orthotopic neobladder, 12 days (IQR 11–14) for W‐shaped orthotopic neobladder, and 8 days (IQR 7–11) for cutaneous ureterostomy (*p* < 0.001).

Table S2 presents postoperative complications and oncological outcomes by urinary diversion type. Although overall rates of early and late complications were comparable among groups, several diversion‐specific differences were observed. Ostomy obstruction, a Clavien–Dindo grade III complication, occurred only after Bricker ileal conduit or cutaneous ureterostomy, with a higher incidence in the Bricker group. Orthotopic neobladder–specific complications, including bladder stones and uracratia, were confined to neobladder reconstructions, predominantly the U‐shaped type (*p* < 0.001). Late urinary tract infection and kidney stone were significantly more frequent in the cutaneous ureterostomy group (*p* = 0.002 and *p* = 0.044). Anxiety and depressive disorders (grade I) were also more common in patients undergoing cutaneous ureterostomy. In contrast, tumor recurrence and distant metastasis rates were low and did not differ significantly among diversion groups.

**FIGURE 1:**
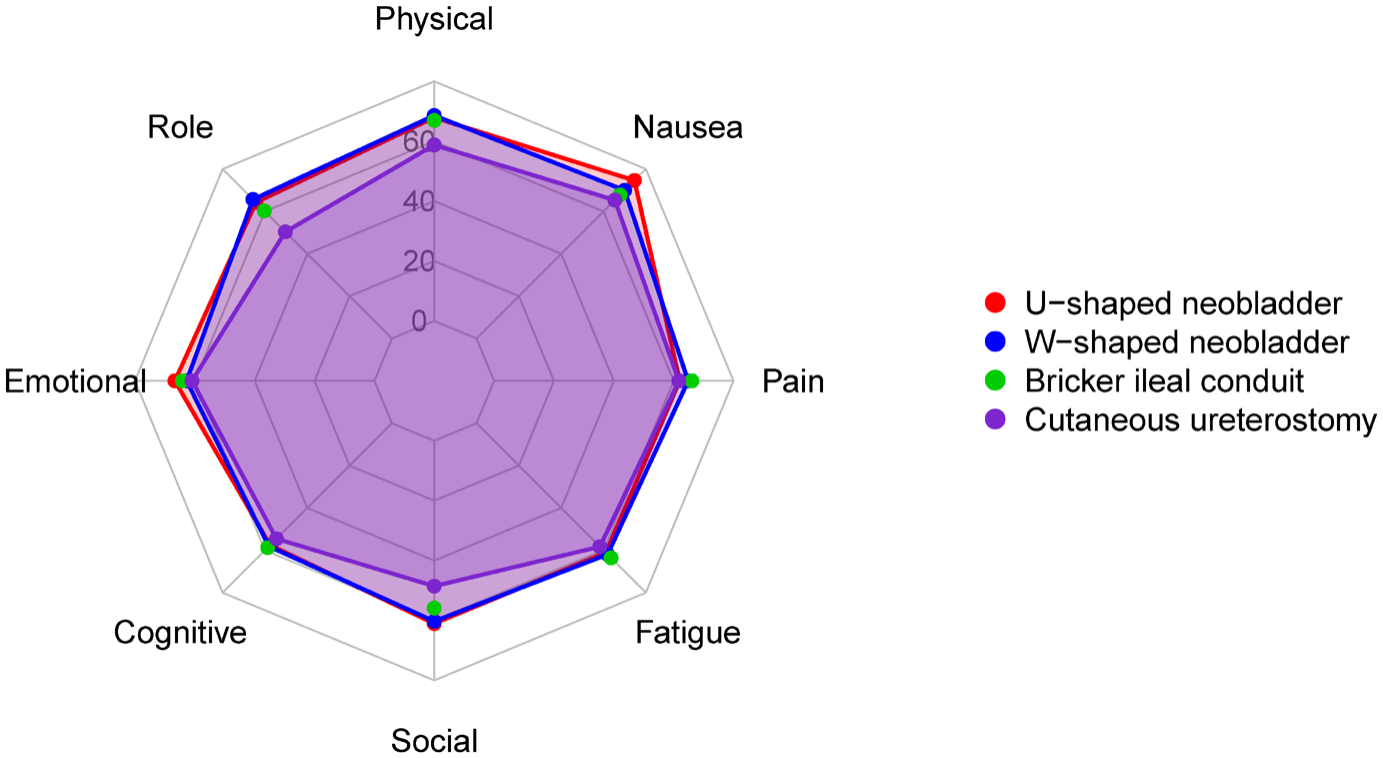
Radar chart of EORTC QLQ‐C30 scores at 1‐year follow‐up for patients undergoing different urinary diversion methods after radical cystectomy.

As shown in Figure 1, at the 1‐year follow‐up after RC, patients who underwent orthotopic neobladder reconstruction reported better overall QoL compared to those with Bricker ileal conduit or cutaneous ureterostomy. Functional domain scores were generally higher, and symptom burdens lower in the neobladder groups. Notably, the W‐shaped orthotopic neobladder subgroup exhibited slightly superior outcomes. In contrast, patients with cutaneous ureterostomy demonstrated the lowest QoL scores across most dimensions.

### 3.3 Time‐Dependent Transitions and Risk Factors for Postoperative Complications

**FIGURE 2:**
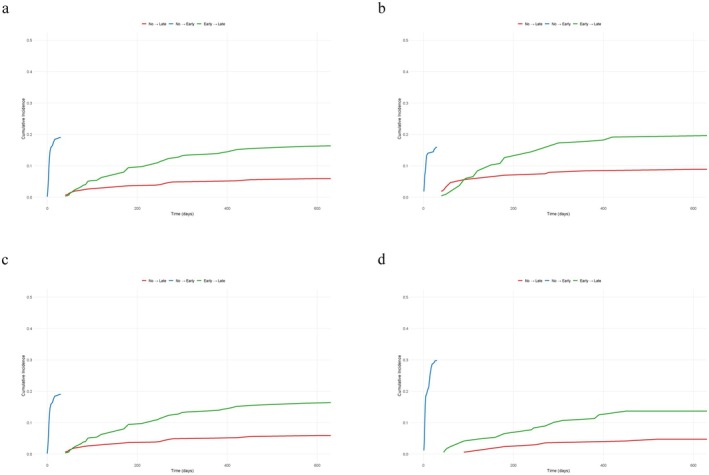
Cumulative probability of transitioning between postoperative complication states following radical cystectomy. (a) All patients; (b) Patients with ureterostomy; (c) Patients with Bricker ileal conduit; (d) Patients with orthotopic neobladder.

Figure 2 illustrates the cumulative incidence of postoperative complications stratified by transition type. The cumulative incidence for the transition from no complications to early complications exhibits a rapid initial increase before reaching a plateau. In contrast, the transition from no complications to late complications shows a gradual initial rise, followed by an accelerated increase at a later stage. The transition from early complications to late complications demonstrates a steep early rise that subsequently stabilizes. Subgroup analyses (Figure 2b–d) showed similar trends, with ureterostomy and Bricker ileal conduit groups displaying comparable early complication rates but varying late complication risks. Notably, the orthotopic neobladder group exhibited the lowest incidence of late complications. These findings suggest that early complications are common across all diversion types, but the risk of late complications varies by reconstruction method.

Multistate Cox regression analysis (Table 2) identified key factors influencing transitions between postoperative complication states after RC. Older age increased the risk of progressing from no complications to early (HR = 1.02, *p* = 0.048) and late complications (HR = 1.03, *p* = 0.012). Male gender was protective against both early (HR = 0.54, *p* = 0.007) and late complications (HR = 0.69, *p* = 0.037). Heavy smoking (> 20 cigarettes/day) significantly raised the risk of early to late (HR = 5.89, *p* = 0.017) and no to late complications (HR = 1.93, *p* = 0.044). Longer hospital stay and freelance occupation were linked to a higher risk of early to late complications. Ureterostomy increased risks of early (HR = 1.58, *p* = 0.042) and late complications (HR = 3.12, *p* = 0.016), while U‐shaped orthotopic bladder reconstruction reduced the risk of late complications (HR = 0.54, *p* = 0.004). T4 stage was associated with early complications (HR = 3.26, *p* = 0.005).

**TABLE 2:** Risk factors for transition between postoperative states based on a multi‐state Cox model after radical cystectomy.

Characters	No complication → early complication	*p*	Early complication → late complication	*p*	No complication → late complication	*p*
Age	1.02 (1.00–1.06)	0.048	1.00 (0.97–1.04)	0.802	1.03 (1.00–1.08)	0.012
BMI	0.98 (0.91–1.06)	0.574	1.01 (0.88–1.16)	0.857	0.96 (0.91–1.02)	0.196
Daily cigarette						
0	Reference		Reference		Reference	
> 0 and ≤ 10	1.19 (0.57–2.49)	0.641	1.54 (0.38–6.19)	0.546	0.89 (0.58–1.36)	0.583
> 10 and ≤ 20	1.61 (0.73–3.57)	0.236	4.83 (1.22–19.07)	0.024	1.36 (0.81–2.27)	0.242
> 20	6.89 (0.16–15.01)	0.289	5.89 (1.36–25.60)	0.017	1.93 (1.00–3.71)	0.044
Daily alcohol consumption						
0 mL	Reference		Reference		Reference	
> 0 and ≤ 50mL	1.23 (0.84–1.82)	0.083	1.15 (0.84–1.52)	0.357	1.25 (0.72–2.32)	0.473
> 50 and ≤ 200 mL	1.61 (0.70–2.62)	0.588	1.28 (0.76–1.75)	0.237	1.18 (0.84–1.96)	0.442
> 200 mL	1.58 (0.67–2.44)	0.482	1.44 (0.71–1.82)	0.128	1.29 (1.04–1.63)	0.028
EBL	1.00 (1.00–1.00)	0.624	1.00 (0.99–1.01)	0.726	1.00 (1.00–1.00)	0.451
Hospital stay after surgery	0.99 (0.96–1.03)	0.743	1.05 (1.00–1.09)	0.028	1.00 (0.98–1.02)	0.823
Gender						
Female	Reference		Reference		Reference	
Male	0.54 (0.35–0.85)	0.007	1.93 (0.66–5.68)	0.233	0.69 (0.48–0.98)	0.037
Occupation						
Cadre	Reference		Reference		Reference	
Doctor	0.54 (0.07–4.38)	0.562	7.58 (0.41–140.70)	0.174	0.87 (0.24–3.18)	0.832
Farmer	1.14 (0.48–2.73)	0.762	4.45 (0.53–37.34)	0.169	1.14 (0.61–2.14)	0.673
Freelancer	1.47 (0.43–5.08)	0.543	1.56 (1.13–2.29)	0.014	1.42 (0.59–3.43)	0.432
Staff	0.72 (0.31–1.66)	0.446	3.27 (0.41–25.90)	0.262	0.81 (0.44–1.48)	0.488
Teacher	0.59 (0.12–2.83)	0.506	2.92 (0.17–49.54)	0.458	1.05 (0.38–2.86)	0.931
Worker	1.30 (0.60–2.84)	0.510	1.97 (0.24–16.10)	0.528	1.16 (0.65–2.05)	0.611
Smoking history	1.65 (0.68–2.37)	0.255	1.74 (0.55–5.51)	0.348	0.96 (0.59–1.58)	0.878
Drinking history	1.42 (0.20–2.91)	0.375	3.01 (0.59–7.05)	0.082	1.92 (0.65–3.17)	0.994
Surgical approach						
Laparoscopy	Reference		Reference		Reference	
Robotic	1.04 (0.68–1.60)	0.841	1.25 (0.58–2.71)	0.566	0.94 (0.68–1.30)	0.721
Urinary tract reconstruction						
Bricker ileal conduit	Reference		Reference		Reference	
U‐shaped Orthotopic bladder	0.77 (0.41–1.43)	0.402	1.00 (0.31–3.20)	0.998	0.54 (0.34–0.86)	0.004
W‐shaped Orthotopic bladder	1.15 (0.42–3.16)	0.779	1.50 (0.27–8.18)	0.641	0.57 (0.26–1.28)	0.175
Ureterostomy	1.58 (1.08–2.54)	0.042	3.12 (1.24–7.87)	0.016	1.34 (0.91–1.98)	0.135
Pathological grade						
Low grade	Reference		Reference		Reference	
High grade	1.09 (0.69–1.74)	0.562	0.67 (0.29–1.58)	0.359	0.96 (0.67–1.36)	0.805
T stage						
T1	Reference		Reference		Reference	
T2	1.26 (0.73–2.16)	0.412	0.92 (0.37–2.26)	0.851	1.11 (0.77–1.61)	0.587
T3	1.33 (0.67–2.64)	0.409	0.87 (0.27–2.79)	0.809	1.06 (0.64–1.75)	0.821
T4	3.26 (1.43–7.45)	0.005	0.96 (0.11–8.43)	0.970	1.47 (0.74–2.92)	0.266

Abbreviations: BMI, body mass index; EBL, estimated blood loss.

### 3.4 Correlation Analysis of Postoperative Complications

**FIGURE 3:**
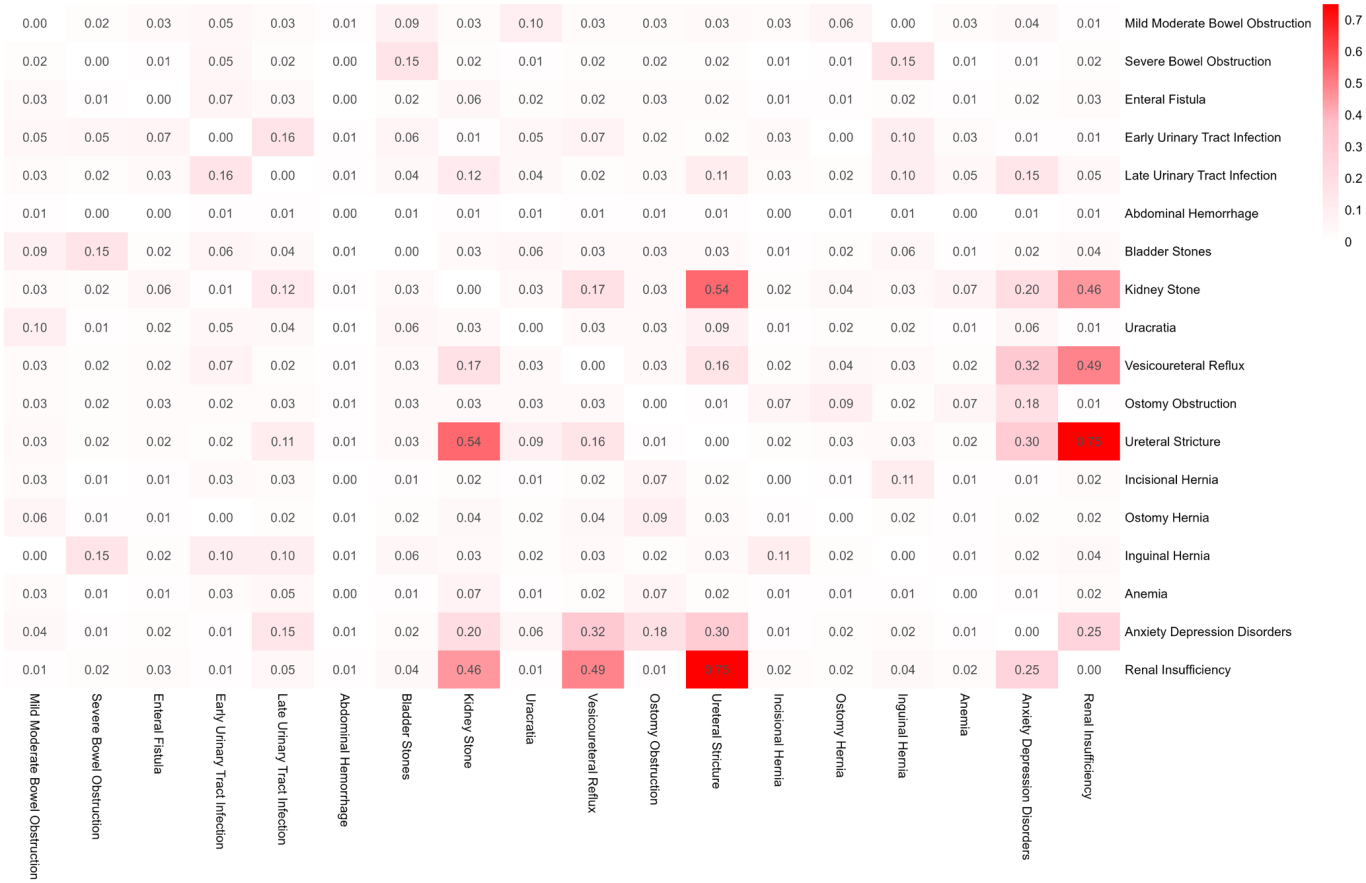
Heatmap of correlations among postoperative complications following radical cystectomy for bladder cancer.

The co‐occurrence heatmap of postoperative complications revealed distinct patterns of association among multiple complication types (Figure 3). Strong correlations were observed between ostomy obstruction and ostomy hernia, as well as between mild to moderate and severe bowel obstruction. Ureteral stricture showed a strong positive correlation with vesicoureteral reflux and renal insufficiency, suggesting a possible shared urological pathophysiology. Additionally, a moderate correlation was noted between early and late urinary tract infections. Anxiety and depressive disorders exhibited weak to moderate associations with several complications, particularly ureteral stricture and urinary tract infections, indicating a potential psychosocial impact of prolonged or recurrent postoperative morbidity. In contrast, bladder and kidney stones showed minimal correlation with other events, suggesting a more isolated clinical course.

### 3.5 Factors Influencing Postoperative Complications

Stepwise multivariate logistic regression identified several independent predictors of postoperative complications following RC (Table 3). Mild to moderate bowel obstruction was less frequent among farmers and workers but increased with higher BMI, moderate smoking (10–20 cigarettes/day), and was reduced by robotic surgery. Early UTI risk was highest in freelancers, staff, and workers, and associated with moderate smoking, greater blood loss, lower BMI, longer hospital stay, and orthotopic neobladder reconstruction. Late UTI was strongly predicted by ureterostomy, heavy smoking (> 20 cigarettes/day), advanced tumor stage (T3–T4), and occupation. Bladder stones correlated with moderate to heavy smoking and prolonged hospitalization. Ureteral stricture risk was highest with ureterostomy and orthotopic neobladder, compounded by longer hospital stay and smoking. Renal insufficiency was independently linked to longer hospitalization, T4 stage, prior abdominal surgery, and urinary tract reconstruction type. Ostomy obstruction risk decreased with robotic surgery and low‐grade pathology, but increased in hypertensive patients. These findings provide valuable insights into risk stratification for postoperative complications in patients with BC.

**TABLE 3:** Stepwise multivariate logistic regression for predictors of postoperative complications following cystectomy.

Complication	Variable	OR (95% CI)	*p*
Mild moderate bowel obstruction	Occupation (Farmer)	0.42 (0.21–0.83)	0.012
	Occupation (Worker)	0.70 (0.47–0.96)	< 0.001
	BMI	1.12 (1.03–1.21)	0.004
	Daily cigarette (> 10 and ≤ 20)	2.16 (1.02–4.39)	0.038
	Surgical approach (Robotic surgery)	0.58 (0.37–0.90)	0.016
Early urinary tract infection	Occupation (Freelancer)	7.75 (2.77–23.92)	< 0.001
	Occupation (Staff)	2.67 (1.19–7.13)	0.028
	Occupation (Worker)	2.40 (1.08–6.37)	0.049
	Daily cigarette (> 10 and ≤ 20)	3.24 (1.88–5.54)	< 0.001
	EBL	1.00 (1.00–1.01)	0.011
	BMI	0.92 (0.86–0.98)	0.010
	Hospital stay	1.03 (1.01–1.06)	0.009
	Urinary tract reconstruction (Orthotopic neobladder)	1.77 (1.14–2.76)	0.011
Late urinary tract infection	Urinary tract reconstruction (Orthotopic neobladder)	0.36 (0.14–0.86)	0.024
	Urinary tract reconstruction (Ureterostomy)	7.26 (3.98–14.10)	< 0.001
	Daily cigarette (> 20)	11.56 (4.92–27.10)	< 0.001
	Hospital stay	1.04 (1.01–1.06)	0.008
	Occupation (Farmer)	2.74 (1.09–8.27)	0.047
	Occupation (Worker)	8.62 (2.58–31.75)	< 0.001
	Occupation (Freelancer)	3.03 (1.12–9.54)	0.039
	T stage (T3)	2.73 (1.40–5.42)	0.003
	T stage (T4)	3.20 (1.24–7.82)	0.012
Bladder stone	Daily cigarette (> 10 and ≤ 20)	7.27 (1.64–36.36)	0.011
	Daily cigarette (> 20)	5.06 (1.28–19.65)	0.018
	Hospital stay	1.11 (1.05–1.19)	< 0.001
Kidney stone	Urinary tract reconstruction (Ureterostomy)	4.45 (2.36–9.05)	< 0.001
	BMI	0.89 (0.80–0.97)	0.010
Vesicoureteral reflux	T stage (T4)	4.41 (1.70–10.95)	0.002
	Daily cigarette (> 20)	2.36 (1.02–4.95)	0.031
	Urinary tract reconstruction (Orthotopic bladder)	0.37 (0.17–0.74)	0.006
Ostomy obstruction	Surgical approach (Robotic)	0.41 (0.22–0.71)	0.002
	Hypertension (Yes)	2.25 (1.26–3.95)	0.005
	Pathological grade (Low grade)	0.51 (0.25–0.97)	0.048
Ureteral stricture	Urinary tract reconstruction (Ureterostomy)	17.46 (7.11–51.51)	< 0.001
	Urinary tract reconstruction (Orthotopic neobladder)	3.59 (1.19–11.90)	0.027
	Hospital stay	1.04 (1.00–1.07)	0.031
	Daily cigarette (> 10 and ≤ 20)	2.74 (1.31–5.59)	0.006
	Daily cigarette (> 20)	3.53 (1.13–10.14)	0.022
Renal insufficiency	Hospital stay	1.06 (1.03–1.09)	< 0.001
	T stage (T4)	5.08 (1.82–13.56)	0.001
	Pathological grade (Low grade)	0.51 (0.27–0.92)	0.031
	Abdominal surgery history (Yes)	1.96 (1.13–3.31)	0.014
	Urinary tract reconstruction (Ureterostomy)	4.40 (2.44–8.41)	< 0.001
	Urinary tract reconstruction (Orthotopic neobladder)	5.38 (1.01–23.70)	0.031

Abbreviations: BMI, Body mass index; EBL, estimated blood loss.

## 4 Discussion

Complications following radical cystectomy evolve along a temporal continuum, with early events such as anastomotic leakage, postoperative ileus, and wound infection often resulting in prolonged hospitalization or re‐intervention, and late complications including ureteroenteric strictures, urolithiasis, and metabolic disturbances requiring long‐term management [15, 16]. These complication patterns vary substantially by urinary diversion type: cutaneous ureterostomy is prone to stoma‐related complications and recurrent pyelonephritis, Bricker ileal conduit more frequently involves bowel obstruction and anastomotic strictures, and orthotopic neobladder is commonly complicated by urinary incontinence, retention, and electrolyte imbalance [17, 18, 19]. Within this context, our single‐center cohort delineates the temporal distribution and clinical characteristics of diversion‐specific complications beyond the index hospitalization, complementing prior population‐based studies limited to in‐hospital outcomes [20, 21]. In parallel, contemporary randomized evidence and updated meta‐analyses suggest that robotic‐assisted radical cystectomy achieves perioperative and oncological outcomes comparable to open surgery while modifying specific perioperative risk profiles, highlighting the evolving surgical landscape in which diversion‐related morbidity should be interpreted [22].

The selection of urinary diversion following RC is influenced not only by oncological considerations but also by patient‐specific factors such as age, comorbidities, renal function, and overall performance status. Consistent with prior reports, patients undergoing cutaneous ureterostomy in our cohort were generally older, exhibited higher Charlson comorbidity index scores, and had poorer preoperative renal function compared with those receiving ileal conduits or orthotopic neobladders [23, 24]. These baseline differences reflect inherent patient selection in real‐world practice and introduce potential selection bias that should be considered when interpreting postoperative complication rates. In particular, patients selected for cutaneous ureterostomy were generally older and more comorbid, and the higher incidence of early complications in this group may therefore be partly attributable to greater preoperative frailty and surgical vulnerability rather than the diversion technique itself. Conversely, candidates selected for orthotopic neobladder reconstruction are typically younger and healthier, which may contribute to lower overall complication rates despite increased surgical complexity. Although multivariable adjustment was performed, residual confounding related to unmeasured factors such as frailty, functional status, and clinical decision‐making cannot be fully excluded; therefore, comparisons across diversion types should be interpreted as associative rather than causal.

Our findings elucidate distinct complication profiles associated with different urinary diversion techniques post‐cystectomy. The cutaneous ureterostomy group experienced the highest incidence of upper UTI and urolithiasis, aligning with existing literature that attributes these risks to the absence of an anti‐reflux mechanism and direct ureteral exposure, facilitating ascending infections and crystal deposition [25]. Furthermore, these patients more frequently required repeated stent replacements or stoma revisions, which not only impose physical discomfort but also exacerbate psychological distress. The emotional burden associated with a visible urostomy, particularly among elderly or frail individuals, has been well documented and includes diminished social participation and impaired self‐image [26, 27].

Bricker ileal conduits, despite their widespread acceptance, exhibited a higher frequency of bowel‐related complications, notably postoperative ileus and ureteroileal anastomotic strictures, consistent with prior studies [17]. The increased prevalence of ileus may be attributable to extensive bowel manipulation and ileal segment construction. Anastomotic strictures are often secondary to ischemia or tension at the ureteroileal junction, frequently necessitating secondary interventions. By contrast, orthotopic neobladder reconstruction was associated with significantly higher rates of urinary incontinence, retention, and metabolic disturbances. The absorptive properties of ileal mucosa contribute to electrolyte imbalances, particularly hyperchloremic metabolic acidosis, while functional voiding difficulties reflect the complexity of neobladder training and potential detrusor underactivity or outlet obstruction [28, 29]. These findings underscore the importance of patient‐tailored diversion selection and comprehensive postoperative monitoring to promptly identify and manage diversion‐specific complications.

The temporal pattern of complications following RC underscores the need for phase‐specific surveillance. Early complications predominantly occur in the immediate postoperative period and often require urgent clinical attention, prolonged hospitalization, or re‐intervention [30, 31]. In contrast, late complications such as ureteroenteric strictures, urolithiasis, and metabolic abnormalities typically develop insidiously over time, necessitating structured long‐term follow‐up [32]. Our data corroborate this temporal framework and further suggest that incorporating both the timing and progression of complications may provide clinically relevant information beyond cumulative event rates alone. In this context, a dynamic assessment of complication trajectories may assist in preoperative risk stratification and support individualized follow‐up planning according to patient characteristics and diversion type. Recognizing these temporal dynamics facilitates optimized resource allocation and supports the development of tailored surveillance protocols aligned with evolving patient risk profiles throughout the postoperative course. Psychological morbidity is an increasingly recognized component of postoperative outcomes after RC, with marked variation across urinary diversion types. Patients with incontinent diversions, such as cutaneous ureterostomy or Bricker ileal conduits, frequently experience elevated anxiety and depression levels, largely attributable to the presence of a permanent stoma, altered body image, and concerns regarding social stigma or reliance on external appliances. Several studies have reported significantly lower health‐related QOL scores in these patients compared to those with continent orthotopic neobladders, particularly in domains related to emotional well‐being, social functioning, and self‐esteem [33, 34]. In our cohort, this psychological vulnerability was compounded by physical complications, including stoma stenosis, recurrent infections, and repeated interventions, which together prolonged recovery and exacerbated distress. While orthotopic neobladders aim to preserve body integrity, associated complications such as urinary incontinence and retention can also adversely affect mental health and daily functioning. These findings highlight the imperative to integrate routine psychosocial assessments and targeted psychological support within postoperative care pathways. Early identification of at‐risk patients and timely interventions, such as counseling, peer support, and pelvic floor rehabilitation, are essential to improve mental health outcomes and enhance overall recovery and satisfaction with surgical management.

Beyond diversion type, our analysis identified multiple patient‐ and treatment‐related factors that significantly modulate postoperative complication risk. Smoking emerged as a key modifiable risk factor, with moderate to heavy tobacco use correlating with increased incidence of UTI, bladder stones, and ureteral strictures. This is likely mediated by smoking‐induced microvascular injury and impaired tissue healing, consistent with prior studies linking tobacco exposure to higher urologic surgical morbidity [35]. Prolonged hospitalization was independently associated with renal insufficiency, ureteral complications, and bladder stone formation, possibly reflecting greater perioperative complexity and heightened susceptibility to nosocomial infections [36]. Notably, robot‐assisted surgery was associated with reduced risks of bowel obstruction and ostomy‐related complications in our cohort, consistent with evidence linking minimally invasive approaches to lower gastrointestinal morbidity and with recent population‐based data demonstrating fewer adverse in‐hospital outcomes and shorter length of stay after RARC [37, 38]. However, surgical approach was not randomized; robot‐assisted surgery was often selected for technically complex cases, while patient preference and financial considerations also influenced decision‐making. Accordingly, residual confounding cannot be excluded, and these findings should be interpreted as associations. Additionally, occupational and physiological factors influenced complication risk: lower BMI and occupational status were associated with early UTI, whereas elevated BMI and smoking increased the risk of ileus [39]. Advanced tumor stage (T4) and prior abdominal surgery were strong predictors of late renal dysfunction. Collectively, these findings highlight the importance of individualized risk stratification and perioperative optimization to reduce preventable complications after RC.

This study has several limitations. As a single‐center retrospective analysis, it is inherently subject to selection bias and potential unmeasured confounding. Importantly, the choice of urinary diversion was influenced by baseline patient characteristics such as age, comorbidity burden, renal function, and tumor stage, which may have confounded observed differences in postoperative outcomes between diversion groups. In addition, diversion selection may have been influenced by surgeon‐ and institution‐specific practice patterns, introducing residual and temporal confounding. The unequal subgroup sizes may have limited statistical power to detect certain complication patterns, and the absence of patient‐reported outcome measures constrained comprehensive assessment of functional and psychosocial impacts. Furthermore, variability in long‐term follow‐up duration may have led to underestimation of late complications. Accordingly, the findings of this study should be interpreted as associative rather than causal. Prospective, multicenter studies incorporating standardized follow‐up protocols and patient‐centered outcome metrics are warranted to validate and extend our findings.

## 5 Conclusion

This single‐center cohort study delineates diversion‐specific postoperative complication profiles following radical cystectomy. Cutaneous ureterostomy was associated with higher risks of urinary tract infection, renal dysfunction, and psychological morbidity, whereas Bricker ileal conduit was more frequently associated with bowel‐related complications and ureteroenteric strictures. Orthotopic neobladder reconstruction was associated with favorable functional outcomes but a higher incidence of urinary incontinence and metabolic disturbances. In addition to diversion type, advanced age, smoking exposure, prolonged hospitalization, and advanced tumor stage were associated with increased complication risk. Robot‐assisted surgery was associated with fewer gastrointestinal and stoma‐related complications, although these findings should be interpreted in light of non‐randomized surgical selection. Collectively, these findings underscore the importance of individualized diversion selection, risk stratification, and structured long‐term follow‐up to optimize postoperative outcomes and quality of life after radical cystectomy.

## Author Contributions

X.H. and J.M. conceived the study, participated in the investigation, and drafted the manuscript. X.Z. and Y.Y. carried out the data curation and helped to draft the manuscript. Y.H. and Y.W. participated in the data analysis. L.Q. performed data visualization and validation. D.Z. and H.W. carried out supervision and writing – review and editing. H.W. provided funder for this study. All authors read and approved the final manuscript.

## Funding

This study was funded by the Natural Science Foundation of Zhejiang Province (No. ZCLMS25H0501, to HBW).

## Ethics Statement

This study was approved by the Ethics Committee of Zhejiang Provincial People's Hospital. All patients signed informed consent prior to treatment. All procedures were conducted in accordance with the Declaration of Helsinki and its amendments.

## Conflicts of Interest

The authors declare no conflicts of interest.

## Supporting information


**Table S1:** Tumor‐related pathological and perioperative characteristics stratified by type of urinary tract reconstruction.
**Table S2:** Postoperative complications and oncological outcomes stratified by type of urinary tract reconstruction after radical cystectomy.
**Figure S1:**. Flow diagram of patient selection and study cohort formation.

## Data Availability

The data that support the findings of this study are available from the corresponding author upon reasonable request.
