# Supplementary material for: Urinary Diversion–Specific Morbidity After Radical Cystectomy: A Ten‐Year Institutional Experience
Source: Cancer Med. 2026 Mar 15;15(3):e71684. doi: 10.1002/cam4.71684 (PMC13093702; doi:10.1002/cam4.71684)
Supplement: Supplementary file 1 — Table S1: Tumor‐related pathological and perioperative characteristics stratified by type of urinary tract reconstruction. Table S2: Postoperative complications and oncological outcomes stratified by type of urinary tract reconstruction after radical cystectomy. Figure S1:. Flow diagram of patient selection and study cohort formation. [file CAM4-15-e71684-s001.docx]

**Supplementary Table 1.** Tumor-Related Pathological and Perioperative Characteristics Stratified by Type of Urinary Tract Reconstruction

| Characters | Bricker ileal conduit | U-shaped orthotopic neobladder | W-shaped orthotopic neobladder | Cutaneous ureterostomy | *P* |
| --- | --- | --- | --- | --- | --- |
| Surgical approach (%) |  |  |  |  | 0.314 |
| Laparoscopy | 123 (64.1) | 84 (60.9) | 18 (60.0) | 149 (69.6) |  |
| Robot-assisted laparoscopy | 69 (35.9) | 54 (39.1) | 12 (40.0) | 65 (30.4) |  |
| Pathological_grade (%) |  |  |  |  | 0.378 |
| Low grade | 42 (21.9) | 41 (29.7) | 9 (30.0) | 53 (24.8) |  |
| High grade | 150 (78.1) | 97 (70.3) | 21 (70.0) | 161 (75.2) |  |
| With prostate cancer (%) ^f^ | 12 (6.2) | 8 (5.8) | 0 (0) | 20 (9.3) | 0.151 |
| ISUP (%) |  |  |  |  | 0.181 |
| 1 | 4 (30.8) | 2 (20.0) | 0 (0.0) | 8 (50.0) |  |
| 2 | 2 (15.4) | 3 (30.0) | 2 (100.0) | 3 (18.8) |  |
| 3 | 4 (30.8) | 5 (50.0) | 0 (0.0) | 1 (6.2) |  |
| 4 | 2 (15.4) | 0 (0.0) | 0 (0.0) | 2 (12.5) |  |
| 5 | 1 (7.7) | 0 (0.0) | 0 (0.0) | 2 (12.5) |  |
| Secondary bladder cancer (%) | 3 (1.6) | 2 (1.4) | 0 (0.0) | 5 (2.3) | 0.913 |
| Pathological type (%) |  |  |  |  | 0.348 |
| Urothelial carcinoma | 189 (98.4) | 131 (94.0) | 30 (100) | 204 (95.2) |  |
| squamous cell carcinoma | 1 (0.5) | 5 (3.6) | 0 (0) | 1 (0.5) |  |
| Adenocarcinoma | 2 (1.1) | 0 (0) | 0 (0) | 5 (2.3) |  |
| small cell carcinoma | 0 (0) | 1 (0.7) | 0 (0) | 3 (1.4) |  |
| spindle cell carcinoma | 0 (0) | 1 (0.7) | 0 (0) | 1 (0.5) |  |
| Nerve invasion (%) | 30 (15.6) | 25 (18.1) | 4 (13.3) | 40 (18.7) | 0.822 |
| vascular invasion (%) ^d^ | 36 (18.8) | 18 (13.0) | 9 (30.0) | 41 (19.2) | 0.137 |
| pT (%) |  |  |  |  | 0.547 |
| T1 | 50 (26.0) | 38 (27.5) | 11 (36.7) | 42 (19.6) |  |
| T2 | 99 (51.6) | 76 (55.1) | 15 (50.0) | 126 (58.9) |  |
| T3 | 33 (17.2) | 21 (15.2) | 3 (10.0) | 36 (16.8) |  |
| T4 | 10 (5.2) | 3 (2.2) | 1 (3.3) | 10 (4.7) |  |
| pN (%) |  |  |  |  | 0.633 |
| N0 | 182 (94.8) | 133 (96.4) | 29 (96.7) | 204 (95.3) |  |
| N1 | 6 (3.1) | 5 (3.6) | 0 (0.0) | 7 (3.3) |  |
| N2 | 1 (0.5) | 0 (0.0) | 0 (0.0) | 2 (0.9) |  |
| N3 | 3 (1.6) | 0 (0.0) | 1 (3.3) | 1 (0.5) |  |
| pM (%) |  |  |  |  |  |
| M0 | 190 (99.0) | 138 (100.0) | 30 (100.0) | 211 (98.6) | 0.582 |
| M1 | 2 (1.0) | 0 (0.0) | 0 (0.0) | 3 (1.4) |  |
| Hemoglobin decrease (g/dL) ^e, f^ | 2.30 [1.80, 3.42] | 2.40 [1.90, 3.60] | 2.55 [1.80, 3.17] | 2.00 [1.50, 3.30] | 0.004 |
| Hospital stay after surgery (days, median [IQR]) ^a, e, f^ | 11.00 [9.00, 13.25] | 12.00 [10.00, 14.00] | 12.00 [11.00, 14.00] | 8.00 [7.00, 11.00] | <0.001 |

^a^ indicates a statistically significant difference between Bricker ileal conduit and U-shaped orthotopic neobladder (P < 0.05). ^b^ indicates a statistically significant difference between Bricker ileal conduit and W-shaped orthotopic neobladder (P < 0.05). ^c^ indicates a statistically significant difference between cutaneous ureterostomy (P < 0.05). ^d^ indicates a statistically significant difference between U-shaped orthotopic neobladder and W-shaped orthotopic neobladder (P < 0.05). ^e^ indicates a statistically significant difference between U-shaped orthotopic neobladder and cutaneous ureterostomy (P < 0.05). ^f^ indicates a statistically significant difference between W-shaped orthotopic neobladder and cutaneous ureterostomy (P < 0.05).

**Supplementary Table 2.** Postoperative Complications and Oncological Outcomes Stratified by Type of Urinary Tract Reconstruction After Radical Cystectomy

| Characters | Bricker ileal conduit | U-shaped orthotopic neobladder | W-shaped orthotopic neobladder | Cutaneous ureterostomy | *P* |
| --- | --- | --- | --- | --- | --- |
| Early postoperative complications (%) ^a,e^ | 31 (16.1) | 35 (25.4) | 6 (20.0) | 35 (16.4) | 0.136 |
| Late postoperative complications (%) ^c^ | 40 (20.8) | 36 (26.1) | 7 (23.3) | 65 (30.4) | 0.121 |
| Mild or moderate bowel obstruction (Clavien-Dindo grade II, %) | 14 (7.3) | 13 (9.4) | 4 (13.3) | 13 (6.1) | 0.384 |
| Severe bowel obstruction (Clavien-Dindo grade III, %) | 1 (0.5) | 2 (1.4) | 0 (0.0) | 0 (0.0) | 0.309 |
| Enteral fistula (Clavien-Dindo grade III, %) | 1 (0.5) | 4 (2.9) | 0 (0.0) | 0 (0.0) | 0.292 |
| Abdominal hemorrhage (Clavien-Dindo grade III, %) | 1 (0.5) | 0 (0.0) | 0 (0.0) | 0 (0.0) | 0.627 |
| Early urinary tract infection (Clavien-Dindo grade II, %) | 16 (8.3) | 21 (15.2) | 2 (6.7) | 21 (9.8) | 0.221 |
| Late urinary tract infection (Clavien-Dindo grade II, %) ^c, e,f^ | 6 (3.1) | 4 (2.9) | 1 (3.3) | 24 (11.2) | 0.002 |
| Ostomy obstruction (Clavien-Dindo grade III, %) ^a, e^ | 15 (7.8) | 0 (0.0) | 0 (0.0) | 10 (4.7) | 0.002 |
| Inguinal hernia (Clavien-Dindo grade III, %) | 6 (3.1) | 2 (1.4) | 1 (3.3) | 4 (1.9) | 0.591 |
| Anemia (Clavien-Dindo grade II, %) | 4 (2.1) | 0 (0.0) | 0 (0.0) | 1 (0.5) | 0.284 |
| Anxiety depression disorders (Clavien-Dindo grade I, %) ^e,f^ | 3 (1.6) | 1 (0.7) | 0 (0.0) | 9 (4.2) | 0.163 |
| Orthotopic neobladder bladder stones (Clavien-Dindo grade III, %) ^a, b, e, f^ | 0 (0.0) | 11 (8.0) | 2 (6.7) | 0 (0.0) | <0.001 |
| Kidney stone (Clavien-Dindo grade III, %) ^c, e^ | 4 (2.1) | 2 (1.4) | 1 (3.3) | 14 (6.5) | 0.044 |
| Uracratia (Clavien-Dindo grade II, %) ^a, e^ | 0 (0.0) | 11 (8.0) | 1 (3.3) | 0 (0.0) | <0.001 |
| Vesicoureteral reflux (Clavien-Dindo grade II, %) | 12 (6.2) | 4 (2.9) | 1 (3.3) | 4 (1.9) | 0.092 |
| Ureteral stricture (Clavien-Dindo grade III, %) ^c^ | 2 (1.0) | 4 (2.9) | 2 (6.7) | 12 (5.6) | 0.036 |
| Renal insufficiency (Clavien-Dindo grade IV, %) ^c^ | 5 (2.6) | 8 (5.8) | 2 (5.0) | 16 (7.5) | 0.074 |
| Ostomy hernia (Clavien-Dindo grade III, %) | 5 (2.6) | 0 (0.0) | 0 (0.0) | 5 (2.3) | 0.264 |
| Tumor recurrence (%) | 8 (4.2) | 7 (5.1) | 1 (3.3) | 8 (3.7) | 0.936 |
| Distant metastasis (%) | 9 (4.7) | 4 (2.9) | 3 (10.0) | 7 (3.3) | 0.273 |
| Pulmonary metastasis (%) ^f^ | 2 (1.0) | 2 (1.4) | 2 (6.7) | 1 (0.5) | 0.072 |
| Lymph node metastasis (%) | 7 (3.6) | 4 (2.9) | 2 (6.7) | 5 (2.3) | 0.477 |
| Rectal metastasis (%) | 1 (0.5) | 0 (0.0) | 0 (0.0) | 0 (0.0) | 0.627 |
| Hepatic metastasis (%) | 5 (2.6) | 2 (1.4) | 0 (0.0) | 3 (1.4) | 0.835 |
| Bone metastasis (%) | 2 (1.0) | 0 (0.0) | 1 (3.3) | 2 (0.9) | 0.309 |
| Abdominal wall metastasis (%) | 2 (1.0) | 0 (0.0) | 0 (0.0) | 0 (0.0) | 0.271 |
| Uterine metastasis (%) | 0 (0.0) | 0 (0.0) | 0 (0.0) | 1 (0.5) | 1.000 |

^a^ indicates a statistically significant difference between Bricker ileal conduit and U-shaped orthotopic neobladder (P < 0.05). ^b^ indicates a statistically significant difference between Bricker ileal conduit and W-shaped orthotopic neobladder (P < 0.05). ^c^ indicates a statistically significant difference between cutaneous ureterostomy (P < 0.05). ^d^ indicates a statistically significant difference between U-shaped orthotopic neobladder and W-shaped orthotopic neobladder (P < 0.05). ^e^ indicates a statistically significant difference between U-shaped orthotopic neobladder and cutaneous ureterostomy (P < 0.05). ^f^ indicates a statistically significant difference between W-shaped orthotopic neobladder and cutaneous ureterostomy (P < 0.05).


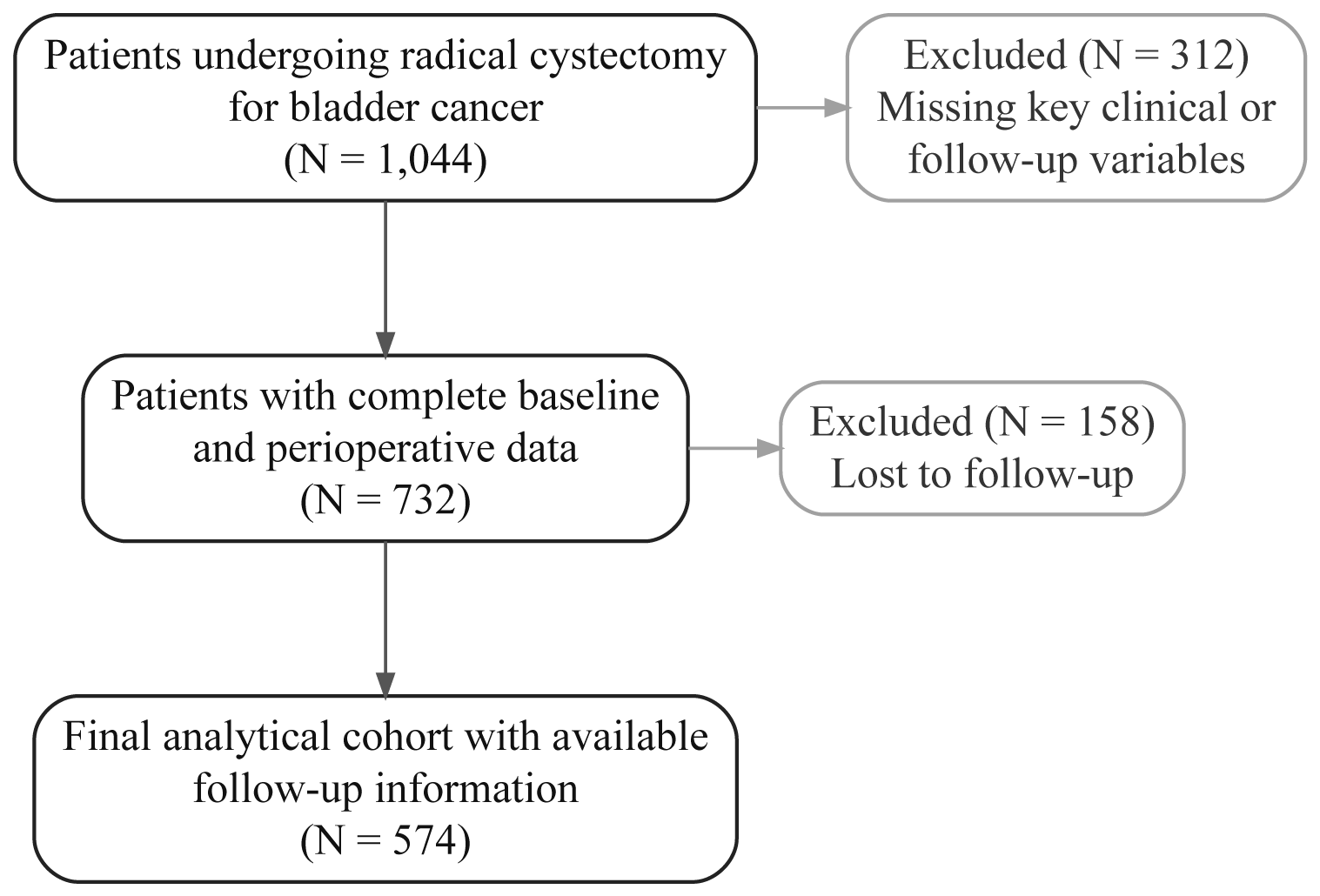


**Figure S1**. Flow diagram of patient selection and study cohort formation
